# Effect of Different Intensities of Aerobic Exercise Combined with Resistance Exercise on Body Fat, Lipid Profiles, and Adipokines in Middle-Aged Women with Obesity

**DOI:** 10.3390/ijerph20053991

**Published:** 2023-02-23

**Authors:** Du-Hwan Oh, Jang-Kyu Lee

**Affiliations:** Department of Exercise Prescription and Rehabilitation, Dankook University, Cheonan 31116, Republic of Korea

**Keywords:** combined exercise, exercise intensity, body fat, lipids profiles, adipokines

## Abstract

We aimed to investigate the effect of different intensities of aerobic exercise (VO_2_max: 50% vs. 80%) on body weight, body fat percentage, lipid profiles, and adipokines in obese middle-aged women after 8 weeks of combined aerobic and resistance exercise. The participants included 16 women aged >40 years with a body fat percentage of ≥30%; they were randomly assigned to the resistance and either moderate (RME, 50% VO_2_max, 200 kcal [*n* = 8]) or vigorous aerobic exercise groups (RVE, 80% VO_2_max, 200 kcal [*n* = 8]), respectively. After 8 weeks of exercise, we observed that body weight and body fat percentage decreased significantly in both groups (*p* < 0.01). The total cholesterol (*p* < 0.01) and LDL (*p* < 0.05) levels decreased significantly in the RME group, while triglyceride levels decreased significantly in both groups (*p* < 0.01). The HDL levels tended to increase only slightly in both groups. The adiponectin levels decreased significantly in the RVE group (*p* < 0.05), and the leptin levels decreased significantly in both groups (*p* < 0.05). To prevent and treat obesity in middle-aged women, combined exercise (aerobic and resistance) is deemed effective; additionally, aerobic exercise of moderate intensity during combined exercise could be more effective than that of vigorous intensity.

## 1. Introduction

According to the World Health Organization (WHO), obesity is defined as “abnormal or excessive fat accumulation that presents a risk to health.” Obesity became a global health problem by the end of the 20th century [1]. Obesity refers to the excessive accumulation of fat in the human body due to excessive caloric intake, irregular lifestyle, and lack of physical activity and is a known cause of various adult diseases, such as diabetes, high blood pressure, hyperlipidemia, and cardiovascular disease [2]. Obesity leads to changes in blood lipid profiles, and there is a direct relationship between chronically elevated cholesterol levels (dyslipidemia) and an increased risk of cardiovascular diseases [3,4].

A major breakthrough in the perception of adipose tissue as an endocrine organ was the discovery of adipokines, which are biologically activated substances; leptin was the first such discovery [5,6]. Adiponectin, an adipokine, enhances insulin sensitivity and promotes anti-inflammatory and antifibrotic activities [7]; however, studies show that adiponectin is reduced in patients with obesity and coronary artery disease, suggesting its crucial role in obesity-associated cardiovascular diseases [7,8]. Leptin—an important hormone in the prevention and treatment of obesity—regulates many physiological processes, such as appetite suppression, energy consumption, and non-shivering thermogenesis [9,10]. The circulating levels of leptin are highly proportional to the amount of adipose tissue [11]. Women with a large relative fat mass tend to exhibit two-fold higher leptin levels in circulation when compared with those of men with similar body weight [12]; therefore, the risk of chronic diseases, which is associated with high circulating levels of leptin, is higher in women than in men.

The WHO recommends exercise, which is the most effective modality for the prevention and treatment of obesity [1]; additionally, regular exercise reduces body fat, improves lipid profiles, and changes adipokine levels [13]. In middle-aged women, reduced physical activity may result in lower estrogen secretion and increasing fat mass and central adiposity; these conditions are linked to the development of morbidities, such as type 2 diabetes, hypertension, atherosclerosis, dyslipidemia, and metabolic syndrome [14]. Physical inactivity and lower hormone secretion in middle-aged women can lead to decreased lean mass, muscle strength, and bone mass; in turn, this may cause musculoskeletal diseases, such as sarcopenia, impaired balance and movement, and increasing falls, consequently decreasing the quality of life [15]. Exercise programs for middle-aged women should therefore include resistance exercises that increase lean mass and muscle strength, and combined exercise—aerobic plus resistance exercises—could alter body fat, lipid profiles, and adipokine levels [14]. In previous studies, combined exercises decreased body weight and body fat [16], improved lipid profiles [14,17], and induced positive changes in adiponectin and leptin levels [14,18].

The most effective exercises for the prevention and treatment of obesity should consider some important factors, such as intensity, volume, frequency, and type of exercise [19]. However, the effects of different aerobic exercise intensities with the same volume and frequency have not yet been elucidated. Therefore, the purpose of this study was to investigate the effects of performing combined resistance and aerobic exercise of different intensities (50% VO_2_max vs. 80% VO_2_max) on body fat, lipid profiles, and adipokines in obese middle-aged women after 8 weeks of exercise. This premise upholds that all participants perform the same amount of exercise (daily energy expenditure of 400 kcal per day by the American College of Sports Medicine’s [ACSMs] recommendation; aerobic exercise—200 kcal and resistance exercise—200 kcal) [20].

## 2. Materials and Methods

### 2.1. Subjects

This study was approved by the Research Ethics Committee of Dongguk University (DGU IRB 20200033-1). We included 16 middle-aged women (age > 40 years) with obesity (>30% body fat) and without any previous diagnosis of metabolic disease or other health problems. The participants did not perform any regular physical activity or exercise. The participants were informed of the procedures and signed a document of informed consent before participating. They were instructed to maintain their typical diet pattern throughout the study, and compliance with this instruction was assessed using food questionnaires (1-day recall). The typical diet was based on the daily recommended calorie intake of 2000 kcal for Korean women and comprised foods commonly consumed by Koreans; expert feedback on the diet was provided. The participants were randomly assigned to the resistance and moderate aerobic exercise (RME, 50% VO_2_max + total body resistance exercise [TRX], *n* = 8) and resistance and vigorous aerobic exercise (RVE, 80% VO_2_max + TRX, *n* = 8) groups according to the intensity of exercise. The physical characteristics of the participants are presented in Table 1.

### 2.2. Body Fat Measurement

The body fat was measured by a certified expert in three regions (triceps, front of thigh, and iliac crest) using the skinfold thickness method, and all processes were conducted according to the International Society for Advanced Kinanthropometry. After measurement, body fat was estimated using the formula by Siri [21] and Jackson et al. [22].

### 2.3. Blood Samples and Analysis

Blood samples were obtained from the antecubital vein after a 12-h fast (both before and after 8 weeks of exercise) and collected into vacutainer tubes with EDTA under the same conditions and time periods. The collected blood was centrifuged at 3000 rpm for 10 min and stored in a deep freezer at −70 °C. The total cholesterol levels and the respective fractions were analyzed using enzymatic colorimetric assays (Modular Analytics Co., Manchester, UK).

### 2.4. VO_2_max Measurement

To estimate VO_2_max, a 1-mile (1609 m) walk was performed by the participants wearing a heart rate monitor (Polar Electro, Kempele, Finland); the rating of perceived exertion was checked every minute to adjust the exercise duration and speed during the test. After the test, the VO_2_max per body weight was estimated based on the exercise time and heart rate with the following formula [23]:

VO_2_max (ml/min/kg) = 132.853 − (0.1692 × body mass in kg) − (0.3877 × age) + (6.315 × sex) − (3.2649 × time in min) − (0.1565 × HR) (1)

Sex: man = 1, woman = 0; HR: Heart rate immediately after the end of walking.

### 2.5. Exercise Program

The exercise program used in this study is shown in Table 2 and was performed five times a week by each group. Energy consumption was measured using the Polar heart rate monitor to measure 400 kcal from when the target intensity was reached (daily energy expenditure of 400 kcal per day by the ACSMs recommendation) [20]. The exercise intensity and rating of perceived exertion were continuously supervised, and the exercise speed was adjusted until the end of the exercise. After a 15-min warm-up under expert supervision, the participants ran on a treadmill at 50% VO_2_max in the RME group and 80% VO_2_max in the RVE group to reach a 200-kcal expenditure. The aerobic exercise was directly supervised by an expert so that the exercise intensity remained fixed for each group. The average times of RME and RVE were 45–48 min and 30–33 min, respectively. As the aerobic component was based on caloric expenditure measured through the heart rate response, the time duration of sessions was individualized to each participant. Thereafter, total body resistance exercise (TRX) was performed. The TRX exercises comprised the use of resistance bands to perform various upper body, lower body, and abdominal exercises (Table 2). Before starting the program, a detailed explanation of the movements was given to the participants, and an expert supervised all exercises. TRX was performed at 60–70% of the HRmax in both groups, for a further 200-kcal expenditure [20]. There were no modifications made to the exercises.

### 2.6. Statistical Analysis

All data analyses in this study were conducted using IBM SPSS Statistics ver. 22.0 (IBM, Armonk, New York, NY, USA). The means and standard errors of all measurements were calculated. The sample size for this study was calculated using the G-Power program (University of Dusseldorf, Dusseldorf, Germany). We set the effect size at 0.2, power at 0.9, number of groups at 2, and number of measurements at 2 for two-way analysis of variance (ANOVA). As the study included a human intervention process, specifically during the COVID-19 pandemic, it was particularly difficult to recruit participants and maintain the study, hence the small sample size with a reduced *Z* power. A two-way ANOVA was used to determine the effects of interactions between group (RME vs. RVE) and time (pre- vs. post-) on the measured variables. When there were significant interaction effects, the post-hoc was analyzed using LSD, and the level of significance was set at α = 0.05.

## 3. Results

### 3.1. Body Weight and Body Fat

**Figure 1:**
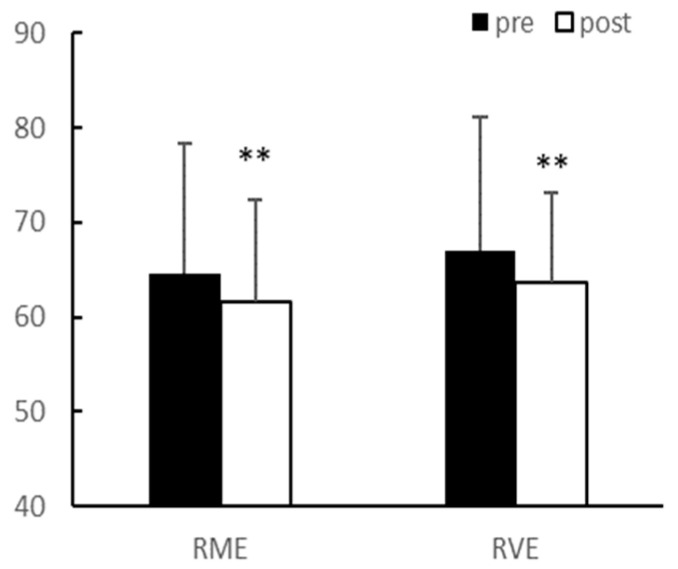
Body weight (kg). Significantly different pre-vs. post-intervention at ** *p* < 0.01.

**Figure 2:**
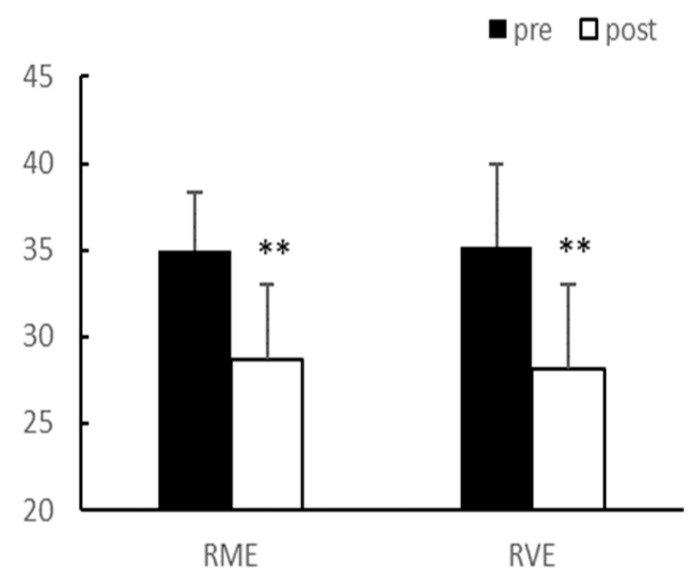
Body fat (%). Significantly different pre-vs. post-intervention at ** *p* < 0.01.

After eight weeks of exercise, there were no significant interaction effects between group and time on body weight and body fat percentage. In the main effect test, although body weight (*p* < 0.01, RME; 64.58 ± 13.69 vs. 61.53 ± 14.18, RVE; 66.95 ± 10.87 vs. 63.64 ± 9.51) and body fat percentage (*p* < 0.01, RME; 34.98 ± 3.39 vs. 28.73 ± 4.75, RVE; 35.23 ± 4.25 vs. 28.15 ± 4.86) significantly decreased after exercise in both groups, there was no difference between the groups (Figure 1 and Figure 2).

### 3.2. Lipid Profiles

**Figure 3:**
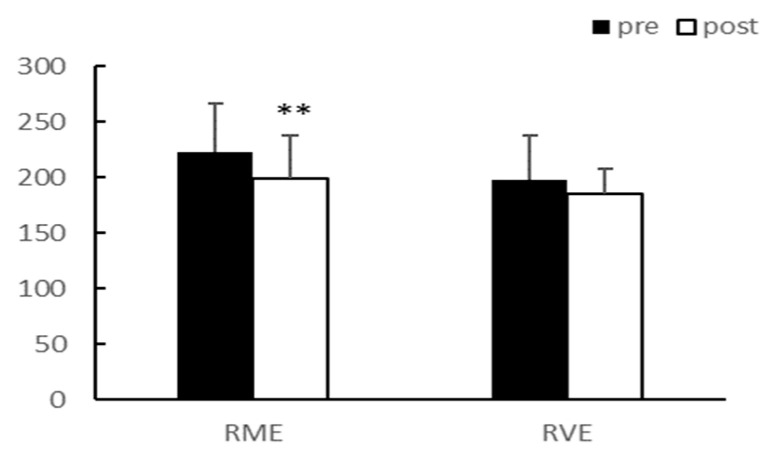
TC (mg/dL). TC, Total cholesterol; ** *p* < 0.01.

**Figure 4:**
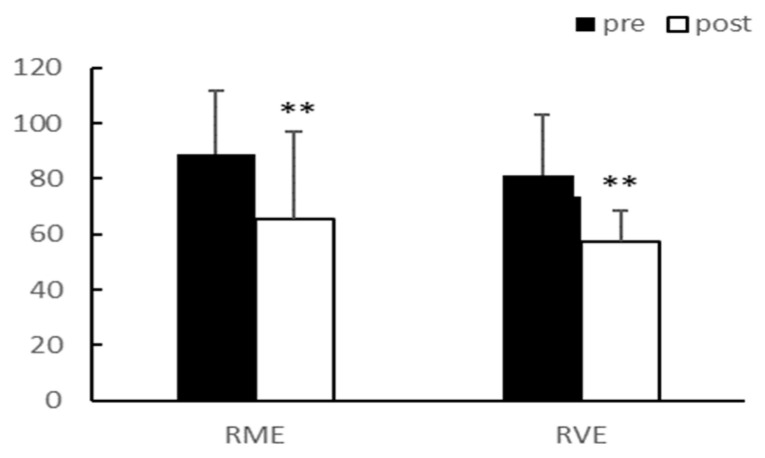
TG (mg/dL). TG, Triglyceride; ** *p* < 0.01.

**Figure 5:**
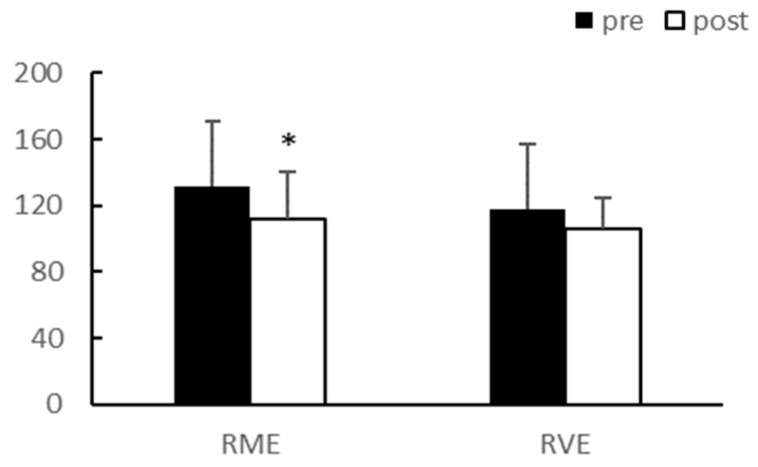
LDL (mg/dL). LDL, Low-density lipoprotein; * *p* < 0.05.

**Figure 6:**
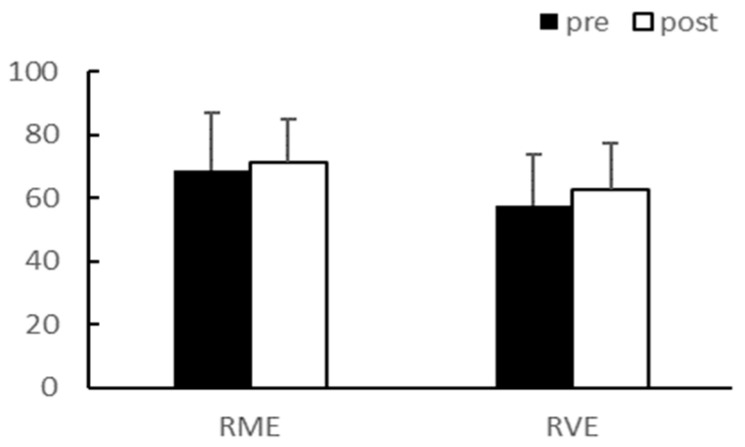
HDL (mg/dL). HDL, High-density lipoprotein.

After eight weeks of exercise, there were no significant interaction effects between group and time for total cholesterol (TC), triglyceride (TG), low-density lipoprotein (LDL), and high-density lipoprotein (HDL). The main effect test results for TC (*p* < 0.01) and LDL (*p* < 0.05) significantly decreased after exercise in the RME group, and TG (*p* < 0.01) significantly decreased after exercise in both groups. Although HDL demonstrated an increasing trend, it was not statistically significant. There were no differences between the groups for any of the variables (Table 3, Figure 3, Figure 4, Figure 5 and Figure 6).

### 3.3. Adipokines

**Figure 7:**
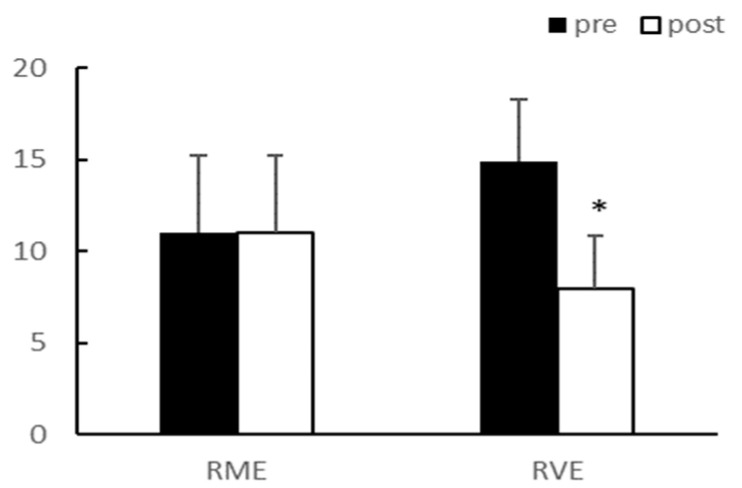
Adiponectin. Significantly different pre vs. post at * *p* < 0.05.

**Figure 8:**
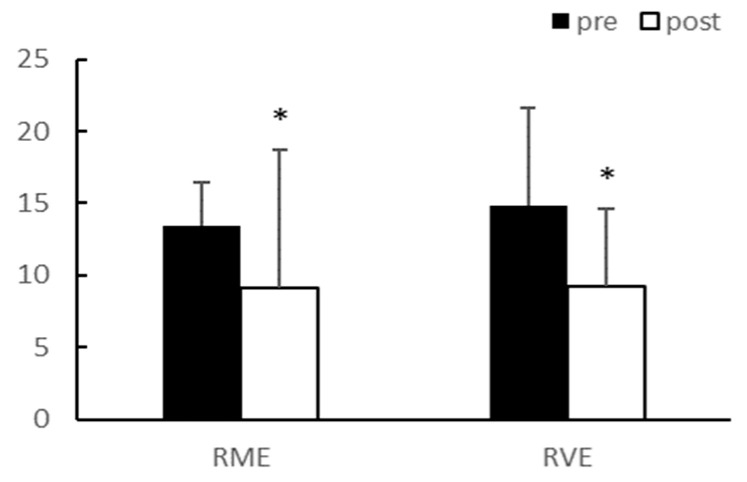
Leptin (mg/dL). Significantly different pre vs. post at * *p* < 0.05.

Adiponectin demonstrated a significant interaction effect between group and time (*p* < 0.05); however, there was no interaction effect on the leptin concentration in the blood. After 8 weeks of exercise, adiponectin levels significantly decreased in the RVE group (*p* < 0.05), and leptin levels significantly decreased in both groups (*p* < 0.05) (Table 4, Figure 7 and Figure 8). The fasting glucose level did not reach statistical significance after exercise, despite exhibiting a decreasing trend.

## 4. Discussion

This study compared the effects of different aerobic exercise intensities for combined exercises (50% VO_2_max + TRX vs. 80% VO_2_max + TRX) on body fat, lipid profiles, and adipokines in middle-aged women. This study revealed important findings regarding the difference in aerobic exercise intensity when combined with resistance exercise and indicated that moderate-intensity aerobic exercise had positive effects on more variables when combined with resistance exercise (TC, TG, LDL, adiponectin, and leptin).

Changes in body weight and body fat are important factors related to the treatment of health problems and diseases [20]. Regular exercise has a positive effect on changes in body weight and body fat [13]. The results of this study are consistent with those of previous studies, which showed that a combination of aerobic and resistance exercise—which increases lipid oxidation, fat-free mass, and resting metabolic rate—significantly reduces weight and body fat [16,24]. Thus, combined exercise may be a more efficient exercise program for decreasing body weight and body fat in obese middle-aged women, regardless of the exercise intensity. However, there was a discrepancy between the expected calorie consumption by the exercise program and the amount of body fat loss. Our findings suggest that adding healthy lifestyle interventions, such as exercise, may motivate participants to choose healthier dietary options, which may further impact calorie expenditure and subsequent weight loss.

Aerobic exercises—such as walking, jogging, and running—are traditionally performed to alter blood lipid profiles, with various results being reported according to exercise intensity [25,26]. O’Donovan et al. [25] controlled the exercise volume to directly assess the impact of aerobic exercise intensity. In their study, participants of the moderate- (60% VO_2_max) and high-intensity (80% VO_2_max) exercise groups completed three 400 kcal sessions weekly for 24 weeks. It was reported that TC and LDL levels only significantly decreased in the high-intensity group (*p* < 0.05). Kraus et al. [26] reported that LDL and TG levels significantly decreased, while HDL levels significantly increased, following high-intensity aerobic exercise (*p* > 05); however, previous studies have reported that moderate-intensity aerobic exercise also significantly decreases blood lipid profiles [27,28]. These results suggest that aerobic exercise is a factor in lipid reduction, regardless of exercise intensity. Although there is limited data on the effects of combined aerobic and resistance exercise, several researchers have suggested that some combined exercises can effectively lower blood lipid profiles and increase HDL. Some previous studies have reported that TC [29], TG [17,30], and LDL [29,30] levels decreased significantly, while HDL [19,31] levels increased significantly after combined exercise (moderate aerobic exercise plus TRX). In this study, the TC levels in the blood decreased significantly in the RME group (*p* < 0.01), while the TG levels decreased significantly in both groups (*p* < 0.01). The LDL levels decreased significantly in the RME group after exercise (*p* < 0.05), and although it did not reach statistical significance, the HDL levels tended to increase slightly in both groups. These results demonstrate that moderate-intensity exercise may activate fat metabolism more effectively and may lead to additional physiological effects, such as increased muscle strength and lean body mass, owing to TRX, which was included in the combined exercise regimen [32].

Although most adipokines secreted by adipocytes have a positive correlation with obesity, adiponectin is negatively correlated with obesity: the levels of adiponectin in the blood decrease with increasing obesity [33]. A decrease in the adiponectin levels in the blood was reported to play an important role in the development of atherosclerosis as it reduced the inhibitory effect on atheromatous production [34]. Additionally, a decrease in adiponectin levels has been reported in patients with obesity [35], type 2 diabetes [36], and cardiovascular disease [34,37].

Adiponectin is closely related to various chronic diseases and is known to be significantly affected by exercise and reductions in body weight and body fat. Lim et al. [38] reported that the levels of adiponectin increased with a decrease in body weight and body fat percentage after combined exercise. However, Hara et al. [39] reported no change in adiponectin levels despite a decrease in body fat after combined exercise, while Paulo et al. [24] and Langleite et al. [40] reported a decrease in adiponectin levels. Although both groups in our study exhibited significantly reduced body weight and body fat after 8 weeks of exercise, the levels of adiponectin decreased significantly in the RVE group, while there were no changes in the RME group. The adiponectin level decreased or exhibited no change because this level is inversely correlated to the adiponectin receptor, which is expressed in the muscle and adipose tissue after exercise [40,41]. Another possible reason is that the increase in catecholamines secreted during exercise and the decreased fasting glucose level after exercise suppressed the gene expression of adiponectin [42,43]. Additionally, there are differences in the degree of weight loss compared to that reported in previous studies. In this study, there was a significant decrease in body weight and body fat; however, the two types of aerobic exercise intensities for combined exercise did not have a positive effect on the increase in blood adiponectin. Therefore, the effect of aerobic exercise intensity combined with resistance training on adiponectin levels remains unclear.

The levels of leptin in the blood increase in proportion to the amount of body fat [11], and it has been reported that blood leptin levels are approximately twice as high in women as those in men with the same body fat (%) [12]. In addition, the risk of various chronic diseases is higher in women. Previous studies reported that leptin levels decreased with a decrease in body weight and body fat after aerobic exercise [44,45]. However, other studies have reported no change in the levels of leptin despite a decrease in body weight and body fat after resistance exercise [46,47] and combined exercise [38]. In the present study, there was a significant reduction in weight and body fat percentage along with leptin levels in both groups after 8 weeks of exercise; this finding is consistent with the results of several previous studies. These results indicate that a decrease in body fat due to exercise is accompanied by a decrease in leptin levels in the blood; this phenomenon is thought to occur due to changes in the fat mass stored in the body through an improved balance of energy and fat metabolism [48]. Additionally, regarding catecholamine changes during exercise, an increase in the activity of norepinephrine induces a decrease in the levels of leptin in the blood by improving the use of fatty acids and reducing leptin resistance [49]. In this present study, effects were observed, but no differences existed between the two exercise programs. Additional studies are needed to examine how the difference in exercise intensity affects other obesity-related factors, such as gut hormones and other adipokines. Although we attempted to manage them, we could not tightly control all individual activities and diets. The limitations of our study include the small sample size, control of calorie intake, and diet composition.

## 5. Conclusions

The purpose of this study was to investigate the effects of different aerobic exercise intensities for combined exercise (50% VO_2_max vs. 80% VO_2_max with TRX) on body weight, body fat, lipid profiles, and adipokines. We reported that weight and body fat decreased significantly in both groups (*p* < 0.01), TC levels in the blood decreased significantly in the RME group (*p* < 0.01), and TG levels decreased significantly in both groups (*p* < 0.01) after exercise. The LDL levels decreased significantly in the RME group (*p* < 0.05), while HDL levels tended to increase slightly in both groups after exercise. The adiponectin levels decreased significantly in the RVE group (*p* < 0.05), and the leptin levels decreased significantly in both groups (*p* < 0.05) after exercise. An interesting finding of this study is that the effect of combined exercise was similar to that of aerobic exercise alone for improvements in body fat, lipid profiles, and adipokine levels. Although, additional physiological effects can be expected due to the resistance exercise component of the combined exercise.

In conclusion, combined aerobic plus resistance exercises could be effective in preventing and treating obesity in middle-aged women. Additionally, aerobic exercise of moderate intensity during combined exercise could be more effective than that of vigorous intensity.

## Figures and Tables

**Table 1:** Participant characteristics.

Group (*n* = 16)	Age (yr)	Height (cm)	Weight (kg)
RME (*n* = 8)	42.25 ± 2.17	159.65 ± 1.88	65.00 ± 3.58
RVE (*n* = 8)	43.00 ± 2.41	163.18 ± 1.46	68.63 ± 3.83

Values are presented as mean ± standard error of the mean (SEM); RME, resistance and moderate aerobic exercise; RVE, resistance and vigorous aerobic exercise.

**Table 2:** Exercise program.

Exercise Type		Exercise Program	Expenditure Calorie
Warming up		Stretching (15 min)	
Main exercise	RME	Treadmill (50% VO_2_max) + TRX TRX program—push up, standing row, Kneeling triceps extension, biceps curl, jump squat, lunge, leg curl, ab slide, reverse lying knee pull	Resistance: 200 kcal Aerobic: 200 kcal
	RVE	Treadmill (80% VO_2_max) + TRX TRX program—push up, standing row, Kneeling triceps extension, biceps curl, jump squat, lunge, leg curl, ab slide, reverse lying knee pull	Resistance: 200 kcal Aerobic: 200 kcal
Cool down		Stretching (15 min)	

TRX, total body resistance exercise.

**Table 3:** Change of lipid profiles.

		Pre-	Post-	*p*-Value		
				Time	Group	Time × Group
TC (mg/dL)	RME	222.63 ± 43.62	199.13 ± 39.61 **	0.004	0.250	0.785
	RVE	198.13 ± 38.24	185.25 ± 22.83			
TG (mg/dL)	RME	88.92 ± 22.85	65.50 ± 21.85 **	0.000	0.218	0.543
	RVE	81.13 ± 31.70	57.40 ± 11.13 **			
LDL (mg/dL)	RME	131.63 ± 39.61	111.50 ± 38.50 *	0.002	0.696	0.696
	RVE	118.13 ± 29.03	106.25 ± 18.51			
HDL (mg/dL)	RME	68.63 ± 18.30	71.00 ± 16.22	0.539	0.380	0.652
	RVE	57.48 ± 13.81	62.85 ± 14.51			

TC, Total cholesterol; TG, Triglyceride; LDL, Low density lipoprotein; HDL, High density lipoprotein. Significantly different pre-vs. post at * *p* < 0.05, ** *p* < 0.01.

**Table 4:** Change of adipokines.

		Pre-	Post-	*p*-Value		
				Time	Group	Time × Group
Adiponectin (µg/mL)	RME	11.01 ± 4.19	11.04 ± 3.41	0.014	0.759	0.013
	RVE	14.89 ± 4.21	7.98 ± 2.87 *			
Leptin (ng/mL)	RME	13.41 ± 3.06	9.11 ± 6.75 *	0.044	0.755	0.775
	RVE	14.84 ± 9.63	9.18 ± 5.48 *			
Fasting glucose (mg/dl)	RME	90.37 ± 4.69	85.88 ± 9.26	0.283	0.521	0.811

Significantly different pre-vs. post at * *p* < 0.05.

## Data Availability

The data are available upon reasonable request to the authors. The data are not publicly available due to the privacy protection of the participants.
